# Abdominal Compartment Syndrome Following Paraesophageal and Diaphragmatic Hernia Repair

**DOI:** 10.14309/crj.0000000000001344

**Published:** 2024-04-26

**Authors:** Shawn A. Brophy, Samuel Minor, Daniel G. French

**Affiliations:** 1 Division of Thoracic Surgery, Department of Surgery, University of Calgary, Calgary, Alberta, Canada; 2 Divisions of General Surgery and Critical Care Medicine, Dalhousie University, Queen Elizabeth II Hospital Halifax, Nova Scotia, Canada; 3 Division of Thoracic Surgery, Department of Surgery, Dalhousie University, Queen Elizabeth II Hospital Halifax, Nova Scotia, Canada

**Keywords:** paraesophageal, hiatal, compartment, surgery, abdominal compartment syndrome

## Abstract

Abdominal compartment syndrome (ACS) is defined as a sustained intra-abdominal pressure ≥ 20 mm Hg, associated with new organ dysfunction. Postoperative ACS can occur following repair of hernias with loss-of-domain. Such loss-of-domain hernias are well described involving incisional hernias, less described involving Bochdalek congenital diaphragmatic hernias (CDHs), but not yet described involving paraesophageal hernias (PEHs) or Morgagni CDHs. We describe a case of postoperative ACS following laparoscopic repair of a PEH and Morgagni CDH. This case demonstrates that prophylactic omentectomy should be considered in select patients undergoing repair of large PEHs or CDHs, as ACS is a rare but potential complication.

## INTRODUCTION

Intra-abdominal hypertension (IAH) is defined as a sustained intra-abdominal pressure (IAP) of ≥12 mm Hg and is directly correlated with mortality in critically ill patients.^1-3^ Abdominal compartment syndrome (ACS) is defined as a sustained IAP ≥20 mm Hg with new associated organ dysfunction and rapidly results in death if not promptly recognized and treated.^4^ Management of ACS initially involves nonoperative measures such as nasogastric (NG) and rectal tube decompression, diuresis, and neuromuscular blockade but ultimately requires surgical decompression if nonoperative treatment is unsuccessful.^5,6^

Loss of domain (LOD) is a term variably defined in the literature but is typically used to describe a large hernia for which much of the intra-abdominal viscera chronically lie outside of the abdominal compartment. Operative repair of LOD hernias is associated with significant physiologic stress, including IAH and ACS.^7^ LOD is well described for abdominal wall hernias but only 2 case reports have been published describing ACS following elective repair of large Bochdalek congenital diaphragmatic hernias (CDHs) in adults.^8,9^ There are currently no examples in the literature of postoperative ACS following repair of LOD PEHs or Morgagni CDHs. The following describes a case of ACS requiring decompressive laparotomy in a patient following elective repair of a symptomatic combined type I PEH and Morgagni CDH.

## CASE REPORT

**Figure 1.**
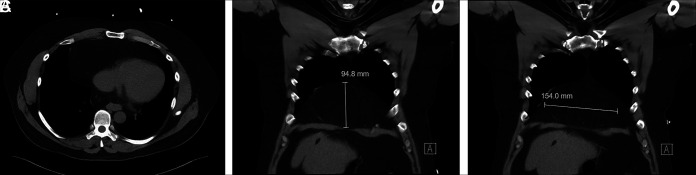
Preoperative cross-sectional imaging demonstrating mediastinal omentum contained within Morgagni congenital diaphragmatic hernia sac in the transverse (A) and coronal (B & C) views.

A 48-year-old man presented to the thoracic surgery clinic with mild anemia, shortness of breath, early satiety, and nocturnal gastroesophageal reflux. Thoracic computed tomography showed a Morgagni CDH and moderate-sized type I PEH containing a significant volume of omentum (Figure 1). Esophagogastroduodenoscopy showed a 6 cm PEH with associated Cameron ulcers. Transthoracic echocardiogram demonstrated normal left ventricular systolic function but revealed a small right atrium and ventricle with diastolic compression of the right atrium, suggesting mass effect from the intrathoracic omentum herniating through the Morgagni CDH. His medical history was significant for obesity (body mass index (BMI) of 37 kg/m^2^) and obstructive sleep apnea (OSA).

The patient underwent a laparoscopic primary suture repair of the Morgagni CDH and PEH with Dor fundoplication. The patient had significant intra-abdominal obesity, and reduction of the hernia contents was difficult despite an insufflation pressure of 15 mm Hg using carbon dioxide. No crural mesh was placed, and no gastropexy was performed during the initial operation. A right chest tube was placed intraoperatively for iatrogenic capnothorax, but apart from the desaturation that prompted this intervention, the patient did not have increased ventilatory pressures or episodes of hemodynamic instability.

In the initial postoperative period, the patient had mild tachycardia of 108 beats per minute, with SpO2 94% on 2 L of supplemental oxygen through nasal prongs, and he was normotensive. The NG tube which had been placed in the OR was unintentionally removed. Over the following 4 hours on postoperative day (POD) 0, the patient became progressively tachypneic, tachycardic, hypotensive, diaphoretic, pale, agitated, and confused, with increasing abdominal pain. Arterial blood gas revealed a mixed metabolic and respiratory acidosis (pH 6.92, pCO2 75, lactate 7.8) with SpO2 97% on 10 L of supplemental oxygen.

Postoperative hemorrhage was suspected, so the patient returned to the OR urgently for an exploratory laparotomy. However, only 700 mL of serosanguineous intra-abdominal fluid was found and no active bleeding. Interestingly, it was noted that the patient's hemodynamic and respiratory status improved immediately with opening of the abdomen. The previous diaphragmatic hernia repairs were intact, but the stomach was grossly distended. The NG tube was reinserted, and the patient's fascia was closed without significant tension. Postoperatively, the patient was sent to the intensive care unit (ICU) intubated for ongoing resuscitation. ACS was suspected based on the intraoperative findings, which was felt to be secondary to gastric distention which developed after unintentional removal of the NG.

The patient stabilized and was weaned from the ventilator within 36 hours. After extubation, however, he required noninvasive ventilation and had respiratory distress when lying supine. On POD 3, the patient developed rapid-onset hypoxia and hypotension. A computed tomography pulmonary angiogram was performed which showed no pulmonary embolus, but the study revealed recurrence of the PEH with an intact Morgagni CDH repair. The patient returned to the OR for a third time for an open omentectomy and gastropexy. During this operation, the crural repair was found to be intact and appropriately snug, suggesting the reherniation through the diaphragmatic hiatus likely occurred due to increased abdominal pressure rather than failure of the crural reapproximation. Postoperatively, the patient was hemodynamically stable, did not require noninvasive ventilation, and was discharged home 4 days after the last operation. At his 3-month follow-up visit, the patient reported resolution of his gastroesophageal reflux and respiratory symptoms, and improving exercise tolerance.

## DISCUSSION

This case describes a rare example of ACS following repair of a Type I PEH and Morgagni CDH. Although there was no IAP measurement, ACS was diagnosed given the significant respiratory and hemodynamic compromise leading to a lactic and respiratory acidosis in the early postoperative period, as well as a hypoxemia, and an ongoing restrictive respiratory pattern after extubation. Alternative diagnoses to explain this patient's symptoms were ruled out, such as hemorrhage, pulmonary thromboembolism, or primary lung disease. ACS following repair of Bochdalek CDHs is described in case reports although no cases of ACS following repair of PEH or Morgagni CDHs have been described.^6,8^

In preoperative assessments of inguinoscrotal hernia repair, scrotal volume to abdominal cavity volume ratio has been described to predict LOD.^10^ Similar techniques have been demonstrated for PEH, and prophylactic omentectomy can be considered to decrease the risk of postoperative ACS.^11^ In this case, an omentectomy at the index operation likely may have avoided the ACS (along with securing the NG tube to prevent unintentional removal) and would have avoided a third surgery for this patient and allowed earlier discharge from ICU. Furthermore, intra-abdominal pressure measurements should be strongly considered in all patients admitted to the ICU following PEH repair, especially those suspected to have IAH or ACS.

The patient described in this case likely had underlying IAH preoperatively. Evidence for this assessment was the patient's elevated BMI, intraperitoneal adiposity, and a spherical rather than pear-shaped abdomen, as well the preoperative echocardiogram findings of a small right atrium and ventricle, with diastolic compression of the right atrium from mass effect of the mediastinal omentum.^12^ The patient returned to the OR on an urgent basis on POD 0 for assumed hemorrhage; however, only 700 cc of serosanguineous fluid was identify with no active bleeding, but the patient improved with opening of his abdominal wall. This case should serve as an example that LOD is possible with a PEH and Morgagni CDH, and if a patient experiences respiratory compromise following surgical repair of these hernias, ACS should be considered as a possible diagnosis. In this case, the omentectomy performed as the third surgery was required to mitigate the increased abdominal pressure that followed reduction of the hernia contents.

## DISCLOSURES

Author contributions: D. French and S. Brophy made contributions to the conception or design of the work and interpreted the data. S. Brophy, S. Minor, and D. French drafted and critically revised the work, gave final approval of the submission, and agreed to be accountable for all aspects of the work. DG French is the article guarantor.

Financial disclosure: None to report.

Informed consent was obtained for this manuscript.
